# Cepharanthine maintains integrity of the blood-brain barrier (BBB) in stroke via the VEGF/VEGFR2/ZO-1 signaling pathway

**DOI:** 10.18632/aging.205678

**Published:** 2024-03-21

**Authors:** Yunfang Yang, Changjiang Li, Sijin Yang, Zhuo Zhang, Xue Bai, Hongmei Tang, Jiang Huang

**Affiliations:** 1 Department of Neurology, The Affiliated Traditional Chinese Medicine Hospital of Southwest Medical University, Luzhou 646000, Sichuan, China; 2 Department of Pharmacology, Southwest Medical University, Luzhou 646099, Sichuan, China; 3 Department of Pharmacy, The Affiliated Traditional Chinese Medicine Hospital of Southwest Medical University, Luzhou 646000, Sichuan, China

**Keywords:** stroke, Cepharanthine, zonula occludens-1 (ZO-1), blood-brain barrier (BBB), hypoxia/reperfusion (H/R)

## Abstract

Dysfunction of tight junctions such as zonula occludens protein-1 (ZO-1)-associated aggravation of blood-brain barrier (BBB) permeability plays an important role in the progression of stroke. Cepharanthine (CEP) is an extract from the plant *Stephania cepharantha*. However, the effects of CEP on stroke and BBB dysfunction have not been previously reported. In this study, we report that CEP improved dysfunction in neurological behavior in a middle cerebral artery occlusion (MCAO) mouse model. Importantly, CEP suppressed blood-brain barrier (BBB) hyperpermeability by increasing the expression of ZO-1. Notably, we found that CEP inhibited the expression of vascular endothelial growth factor (VEGF) and vascular endothelial growth factor receptor 2 (VEGFR2) in the cortex of MCAO mice. Additionally, the results of *in vitro* experiments demonstrate that treatment with CEP ameliorated cytotoxicity of human bEnd.3 brain microvascular endothelial cells against hypoxia/reperfusion (H/R). Also, CEP attenuated H/R-induced aggravation of endothelial permeability in bEND.3 cells by restoring the expression of ZO-1. Further study proved that the protective effects of CEP are mediated by inhibition of VEGF-A and VEGFR2. Based on the results, we conclude that CEP might possess a therapeutic prospect in stroke through protecting the integrity of the BBB mediated by the VEGF/VEGFR2/ZO-1 axis.

## INTRODUCTION

Ischemic stroke is mainly induced by cerebral microcirculation obstruction or destruction resulting from internal or external factors-mediated cerebrovascular vascular blockage, thrombosis, or insufficient blood supply to the brain, which eventually contribute to brain parenchyma cell damage or death, brain dysfunction or damage from acute cerebrovascular disease. Ischemic stroke is difficult to treat due to such characteristics as high incidence and multiple sequelae [1]. It has been reported that destruction of the blood-brain barrier (BBB) after ischemic stroke is an important cause of death due to secondary brain injury, and effective repair of the BBB is one of the main means to treat ischemic stroke. The BBB is composed of brain microvascular endothelial cells (BMECs), an astrocyte terminus, pericytes, and a basement membrane [2], with endothelial cells being its main structure. Endothelial cells and tight junction (TJ) proteins together strictly control BBB permeability [3], which protects the brain from potential neurotoxic substances and facilitates the exchange of nutrients and waste products between the brain and blood to maintain the optimal extracellular environment for nerve function [4]. Damage of the BBB is an important physiopathological basis of ischemic stroke [5] and one of the important causes of death from ischemic stroke. Therefore, the repair of BBB damage may be a possible approach for the treatment of ischemic stroke. Studies have shown that the BBB junction complex is a complicated cellular system with active regulation, consisting TJs and adherent junctions (AJs) [6]. Tight junctions possess highly regulated dynamic structures, consisting of three membrane proteins (Claudin, Occludin, and junction adhesion molecules), cytoplasmic helper proteins (ZO-1, ZO-2, and ZO-3), and Cingulin [7, 8]. Reduced or altered tight junction protein expression in cells will affect cellular permeability and thus impair BBB permeability [9]. It is recently reported that vascular endothelial growth factor (VEGF)/VEGFR2 is involved in the regulation of tight junction protein expression [10]. Thus, VEGF/VEGFR2-mediated BBB permeability might be a promising target for treating ischemic stroke. Ischemic stroke demands an early diagnosis and treatment in a narrow time frame [11]. The standard medical treatment for acute ischemic stroke, such as with intravenous alteplase, is considered for use within 4.5 hours after the onset of symptoms [12, 13]. However, 14 to 27% of strokes occur at night when patients are sleeping, thus symptoms cannot be recognized on time [14]. Such patients are generally excluded from treatment with intravenous alteplase, and only some of them are candidates for mechanical thrombectomy. Therefore, the approaches for preventing and treating ischemic stroke are still being explored.

Cepharanthine (CEP) is an isoquinoline alkaloid and the main component of Tetrandrine tablets, mainly used for the clinical treatment of leukocyte elevation. Previous studies have shown that CEP represses the growth and metastasis of tumor cells, and reduces the resistance of tumor cells to various chemotherapy drugs [15]. Its anti-tumor mechanism is closely related to its regulation of tumor autophagy and apoptosis [16, 17]. Studies have claimed the anti-angiogenesis property of CEP by repressing the expression of VEGF [18]. However, the function of CEP in BBB permeability and ischemic stroke remains unclear. The present study proposes to assess the regulatory function of CEP on middle cerebral artery occlusion (MCAO) mice to explore the potential application of CEP in treating ischemic stroke.

## MATERIALS AND METHODS

### Animal models and treatments

40 C57BL/6J male mice (20-22 g) were sectionalized into 4 groups: Vehicle, CEP, MCAO, and MCAO+CEP (10 mice/group). In the MCAO groups, a surgical filament was inserted into the middle cerebral artery of mice, followed by closing the sutures 2 h later. Then, a reperfusion was allowed for 24 h. In the CEP groups, 1.5 mg/kg CEP was administered into the peritoneal cavity of mice for 3 weeks prior to MCAO modeling [16, 18]. The animals were euthanized after the neurological scores were measured.

### Neurological score

The neurological scoring was utilized to determine the neurological deficit in each animal. Four grades were established: grade 0: neurological deficit was not observed; grade 1: the contralateral forepaw cannot be extended when being grasped on the tail; grade 2: animals moved in circles toward the ipsilateral side; grade 3: animals fell to the contralateral side of brain damage; grade 4: decreased mobility with rare signs of consciousness [19].

### Diffusion of sodium fluorescein assay

Animals were injected with 0.1 mL 10% NaFI, followed by being intraperitoneally injected with 4% chloral hydrate for anesthesia, 45 min later. The chest cavity was then opened and the cannula was intubated to the aorta through the left ventricle, followed by cutting the right atrium to perfuse the normal saline until the clear fluid flowed out of the right atrium. The brain was taken and 0.6 mL phosphate-buffered saline (PBS) homogenate was added into the brain tissue, followed by being centrifuged at 3000 r/min for 5 min and the supernatant was diluted with 20% Trichloroacetic acid (TCA) (1:10) and incubated at 4° C for 24 h. The samples were centrifuged at 10000 r/min for 15 min and the supernatant was collected and then diluted with the same volume of Tris-HCL buffer. Fluorescence intensity (490/514 nm) was measured and the BBB permeability was determined by the amount of NaFI per milligram of brain tissue protein.

### Real-time polymerase chain reaction (PCR)

Trizol reagent was utilized to isolate RNA from brain tissues or cells, followed by being transformed into cDNA using the reverse transcription kit (Takara, Japan). Then the 2×SYBR Green PCR Master Mix kit (Lifeint, China) was utilized to conduct the PCR reaction, and the 2^−ΔΔCt^ method was performed for the calculation of targeted genes [20]. The following primers were used in the study: ZO-1 Forward: 5’-CCCCTCTGTC CAGCTCTTC-3’; Reverse: 5’-CACCGGAGTGATGGTTTTCT-3’; VEGF-A Forward: 5’-TCTACCTCCACCATGCCAAGT-3’, Reverse: 5’-TGCGCTGATA GACATCCATGA-3’; VEGFR2 Forward: 5’-AATTATTGCAGGGGACAGAG-3’, Reverse: 5’-TTCTGGGTATCTTGCACAAAG-3’; β-Actin Forward: 5’-AGGTG ACAGCATTGCTTCTG-3’; Reverse: 5’-GCTGCCTCAACACCTCAAC-3’.

### Immunofluorescence labeling

The immunofluorescence labeling was performed as per a previous study [21]. The brain tissues were embedded in tissue-tek optimal cutting temperature (OCT) compound to be snap-frozen in dry ice and then stored in a -80° C freezer. 7-μm frozen sections were cut using a cryostat-microtome (Leica CM3050S, Leica Biosystems, Germany) at -20° C and were mounted on glass slides. The tissues were then fixed with 4 % paraformaldehyde solution for 10 mins, blocked with 5% BSA for half an hour, and then incubated with primary antibodies against ZO-1 (1:200, Cat# 2847, CST, USA), VEGF (1:200, Cat# sc-365578, Santa Cruz, USA) and VEGFR2 (1:500, Cat# 9698, CST, USA) at room temperature for 2 hours. Then the secondary antibodies were labeled with the corresponding secondary antibodies at room temperature for 1 h: Alexa Fluor 488 AffiniPure goat anti-mouse IgG, (1:800; Cat# 33206ES60, Yeasen, China), and Alexa Fluor plus 555 goat ant-rabbit IgG (H + L) (1:1000; Cat# ab150078, Abcam, USA) were used. Finally, the sections were mounted using the fluorescent mounting medium DAPI G-Fluoromount medium (Southern Biotech, USA), A fluorescence microscope (×40 objective, Nikon Eclipse Ni, Nikon, Japan) coupled with a digitizing camera was used to capture the pictures.

### Human bEnd.3 brain microvascular endothelial cells and cell treatment

Cells were purchased from ATCC (USA) and cultured in a high-glucose Dulbecco’s modified Eagle medium (DMEM) medium containing 10% Fetal Bovine Serum (FBS), which were incubated at 37° C and 5% CO_2_. For the establishment of the H/R model, cells were placed in the anaerobic incubator, followed by introducing 5% CO_2_+95% N_2_ into the incubator. After incubating for 6 h, cells were placed in the incubator with normal conditions and incubated for 24 h.

### 3-(4,5-dimethylthiazol-2-yl)-2,5-diphenyltetrazolium bromide (MTT) assay

Cells were implanted in a 96-well plate (3×10^3^/well) and incubated for 12 h, followed by adding 20 μL 0.5 mg/mL MTT solution. After 4h-incubation at 37° C, the supernatant was disregarded and 200 μL dimethyl sulfoxide (DMSO) was added for 15 mins, followed by measuring the optical density (OD) value at 570 nm using a microplate reader (Tecan, Switzerland).

### Lactate dehydrogenase (LDH) release assay

After treatments, 20 μL supernatant was collected and mixed with 250 μL matrix buffer and 50 μL coenzyme I solution, followed by incubation for 15 min at 37° C. Then 250 μL 2, 4-dinitrophenylhydrazine was introduced for 15 min at 37° C, followed by introducing NaOH to terminate the reaction. Lastly, the OD value was achieved at 440 nm with a microplate reader (Tecan, Switzerland).

### Fluorescein isothiocyanate (FITC)-dextran assay

Cells were planted on the luminal side of filters (Millipore, USA), followed by introducing FITC-dextran (AbMole, USA) into the upper compartment. Following incubation for 1 h, the microplate reader (Tecan, Switzerland) was used to detect the OD value of the solution in the lower chamber at 492/520 nm.

### Trans-endothelial electrical resistance (TEER)

The *in vitro* endothelial permeability was evaluated using the TEER assay with the 1600R ECIS System (Applied Biophysics, Australia) according to the method described previously [22] (Rom et al*.* 2015). The data were expressed with an average of the resistance values (Ω·cm^2^) and the average percent change from baseline TEER (Mean  ± SD).

### Western blotting assay

Proteins were isolated and quantified using the Bicinchoninic acid (BCA) method and loaded to sodium dodecyl sulfate (SDS)-polyacrylamide gel (PAGE) for separation. Then, the proteins in the gel were transferred onto the polyvinylidene fluoride (PVDF) membrane, followed by being blocked using the 5% BSA reagent. Primary antibodies against ZO-1 (1:2000, Cat# 2847, CST, USA), VEGF (1:2000, Cat# sc-365578, Santa Cruz, USA), VEGFR2 (1:1500, Cat# 9698, CST, USA), and β-actin (1:8000, Cat# GTX100313, GeneTex, USA) were then added and incubated for 12 h at 4° C, followed by introducing the anti-rabbit IgG, horseradish peroxidase (HRP)-linked antibody (Cat#7074, CST, USA), anti-mouse IgG, and HRP-linked antibody (Cat# 7076, CST, USA). After 1.5 h incubation, an enhanced chemiluminescence (ECL) solution was added for exposure, followed by quantification of the protein expression using the Image J software [23].

### Statistical analysis

Data were expressed as mean ± standard deviation (S.D.) and the comparison was analyzed using an analysis of variance (ANOVA) method by the software GraphPad Prism 8. The Chi-square test was used as a post-hoc test. P<0.05 was taken as a statistically significant difference.

### Data availability statement

Data will be made available on reasonable request.

## RESULTS

### CEP improved neurological dysfunction in a MCAO mice model

**Figure 1:**
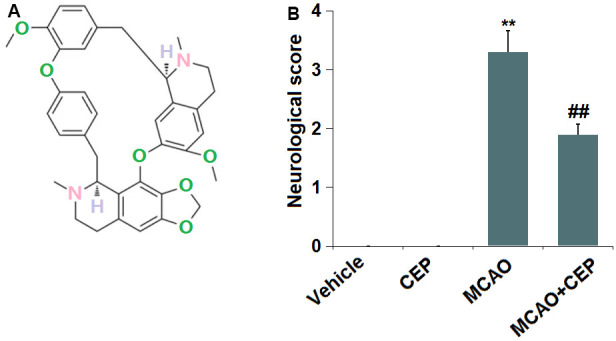
**Cepharanthine (CEP) improved neurological dysfunction in a middle cerebral artery occlusion (MCAO) mice model.** (**A**) Molecular structure of Cepharanthine; (**B**) Neurological score graph of the three experimental groups (**, P<0.01 vs. vehicle group; ##, P<0.01 vs. MCAO group).

The molecular structure of CEP is shown in Figure 1A. 15 mg/kg CEP was injected into the peritoneal cavity of mice for 3 weeks before the MCAO modeling. The neurological score (Figure 1B) in the control and CEP group was 0, while it was elevated to 3.3 in the MCAO group, then declined to 1.9 by treatment with 15 mg/kg CEP, suggesting a protective property of CEP against neurological dysfunction in MCAO mice.

### CEP prevented the increase in BBB permeability in MCAO mice

**Figure 2:**
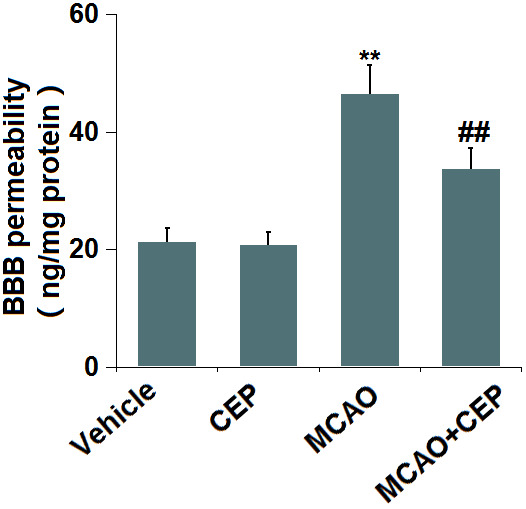
**Cepharanthine (CEP) prevented the increase in blood-brain barrier (BBB) permeability in MCAO mice model.** Blood-brain barrier permeability was measured by diffusion of sodium fluorescein assay (**, P<0.01 vs. vehicle group; ##, P<0.01 vs. MCAO group).

Diffusion of sodium fluorescein assay was used to assess BBB permeability. We found that the BBB permeability (Figure 2) was minorly changed from 21.3 to 20.7 ng/mg protein in CEP-treated normal mice, but was greatly promoted to 46.5 ng/mg protein in MCAO mice. After treatment with 15 mg/kg CEP, the BBB permeability was reduced to 33.7 ng/mg protein, implying an inhibitory effect of CEP on the increased BBB permeability in MCAO mice.

### CEP restored the expression of ZO-1 in the cortex of MCAO mice

**Figure 3:**
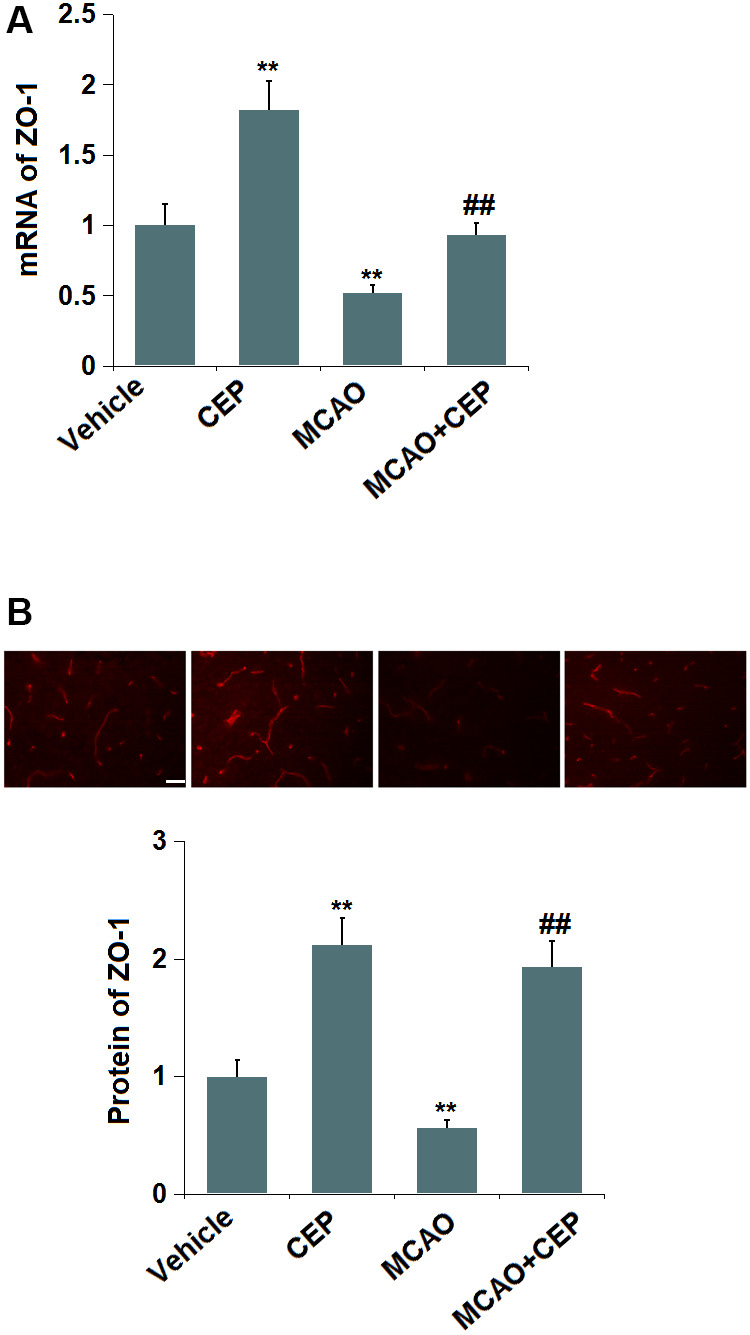
**Cepharanthine (CEP) restored the expression of ZO-1 in the cortex of MCAO mice model.** (**A**) mRNA of ZO-1; (**B**) Protein of ZO-1 as measured using immunostaining. Scale bars, 100 μm (**, P<0.01 vs. vehicle group; ##, P<0.01 vs. MCAO group).

We further checked the impact of CEP on TJ protein level. ZO-1 was found (Figure 3A) significantly upregulated in CEP-treated normal mice, but downregulated in MCAO mice, which was greatly reversed by 15 mg/kg CEP, suggesting a protective effect of CEP on the downregulation of ZO-1 in MCAO mice.

### CEP reduced the expression of VEGF-A and VEGFR2 in the cortex of MCAO mice

**Figure 4:**
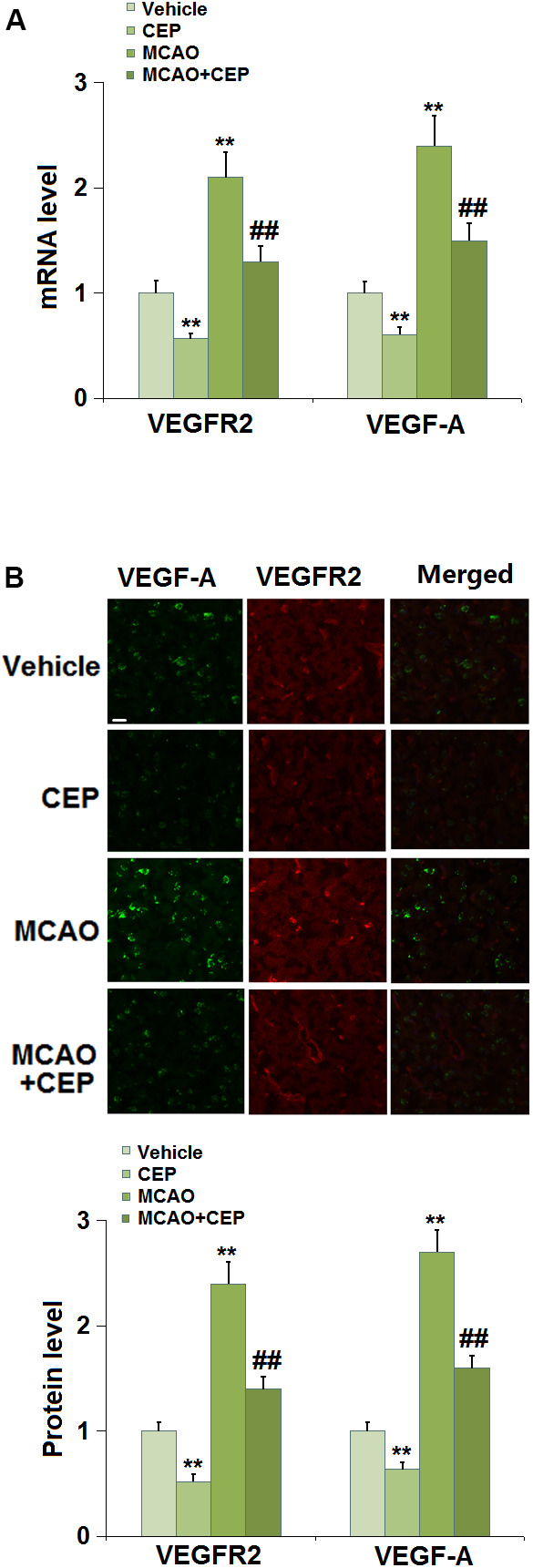
**Cepharanthine (CEP) reduced the expression of VEGF-A and VEGFR2 in the cortex of MCAO mice model.** (**A**) mRNA levels of VEGF-A and VEGFR2; (**B**) Protein levels of VEGF-A and VEGFR2 as measured using immunostaining. Scale bars, 100 μm (**, P<0.01 vs. vehicle group; ##, P<0.01 vs. MCAO group).

The VEGF-A/VEGFR2 axis is reported to regulate the expression of TJ proteins in the BBB [10, 24]. We found that the expression levels of VEGF-A and VEGFR2 (Figure 4A, 4B) were repressed in CEP-treated normal mice and increased in MCAO mice. After the administration of 15 mg/kg CEP to MCAO mice, the expression levels of VEGF-A and VEGFR2 were significantly declined, indicating its inhibitory effect on the VEGF-A/VEGFR2 axis in MCAO mice.

### CEP ameliorated cytotoxicity of bEnd.3 cells against H/R

**Figure 5:**
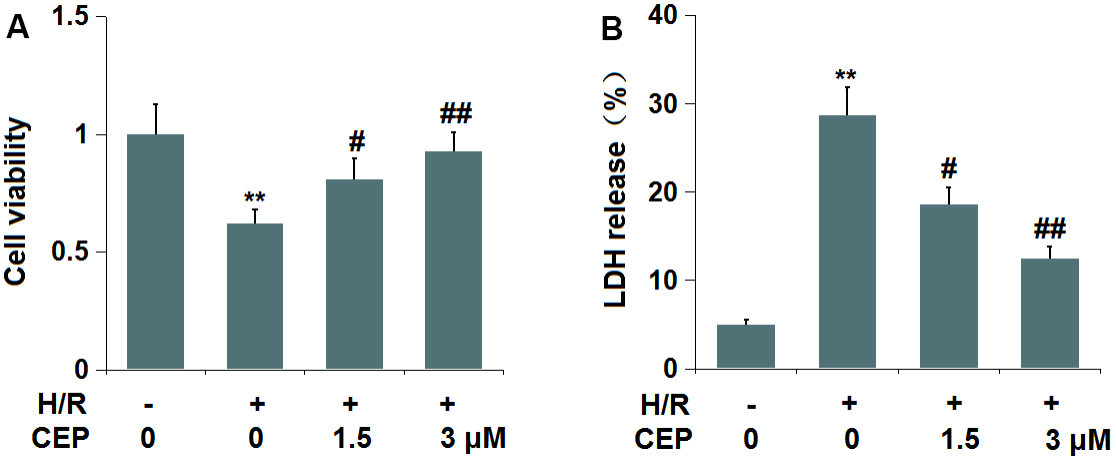
**Cepharanthine (CEP) ameliorated cytotoxicity of human bEnd.3 brain microvascular endothelial cells against hypoxia/reperfusion (H/R).** Cells were exposed to hypoxia/reperfusion condition (6 h/24 h) with or without CEP (1.5, 3 μM). (**A**) Cell viability measured by MTT assay; (**B**) LDH release (**, P<0.01 vs. vehicle group; #, ##, P<0.05, 0.01 vs. H/R group).

To explore the potential mechanism of the protective effects of CEP on stroke, we established an *in vitro* H/R (6 h/24 h) model, followed by treatment with 1.5 and 3 μM CEP. We found that the cell viability (Figure 5A) in H/R-treated cells declined from 100% to 62%, which was elevated to 81% and 93% by 1.5 and 3 μM CEP, respectively. Furthermore, the LDH release in the control, H/R, 1.5 CEP, and 3 μM CEP groups was 5%, 28.7%, 18.6%, and 12.5%, respectively. These data imply that the cytotoxicity in H/R-treated bEnd.3 cells was ameliorated by CEP.

### CEP attenuated H/R-induced aggravation of endothelial permeability in bEnd.3 cells

**Figure 6:**
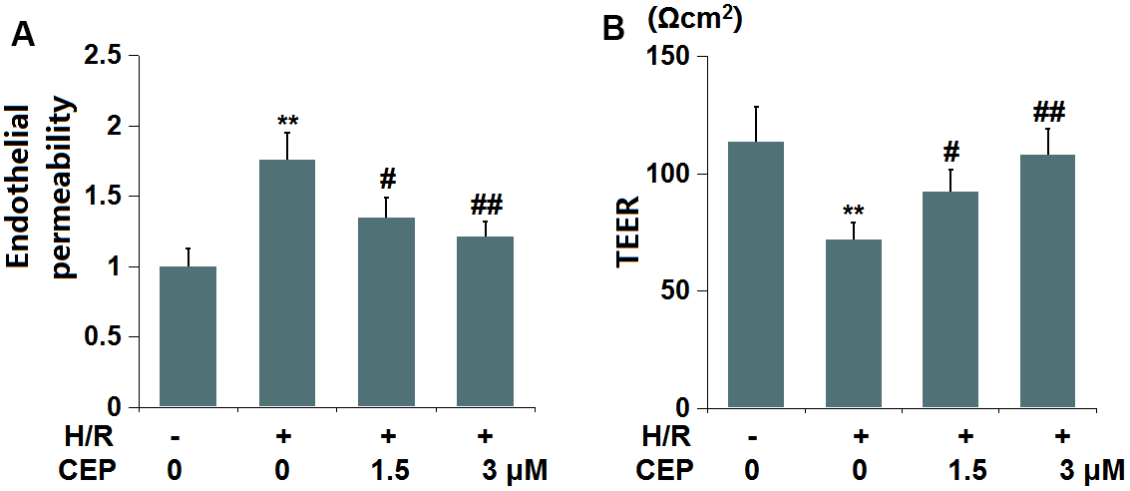
**Cepharanthine (CEP) attenuated hypoxia/reperfusion-induced aggravation of endothelial permeability in brain bEND.3 endothelial cells.** (**A**) Endothelial permeability was measured using FITC-dextran; (**B**) The trans-endothelial electrical resistance (TEER) (**, P<0.01 vs. vehicle group; #, ##, P<0.05, 0.01 vs. H/R group).

We further evaluated the impact of CEP on *in vitro* endothelial permeability. The fluorescence intensity of FITC (Figure 6A) was extremely increased in the H/R group, then reduced by 1.5 and 3 μM CEP. Moreover, the TEER value (Figure 6B) in H/R- treated cells was decreased from 113.5 Ωcm^2^ to 71.8 Ωcm^2^, which was increased to 92.3 Ωcm^2^ and 108.9 Ωcm^2^ by 1.5 and 3 μM CEP, respectively. These data imply the increased endothelial permeability in H/R-challenged bEnd.3 cells was attenuated by CEP.

### CEP restored the expression of ZO-1 in bEnd.3 cells

**Figure 7:**
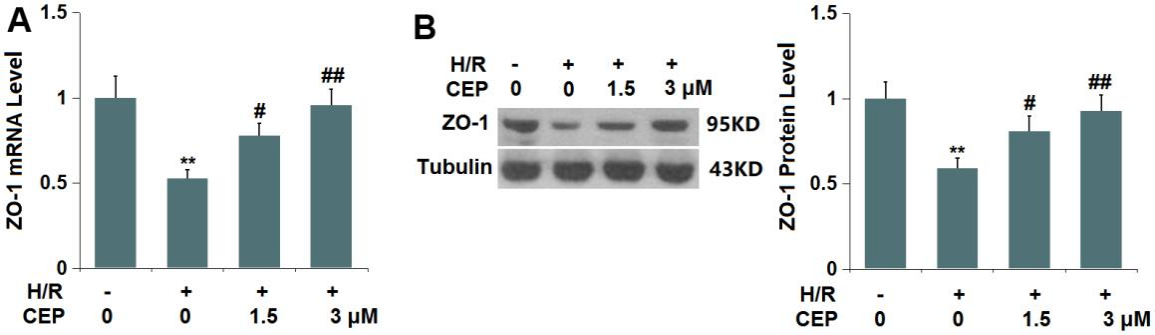
**Cepharanthine (CEP) restored the expression of ZO-1 in brain bEND.3 endothelial cells.** (**A**) mRNA of ZO-1; (**B**) Protein of ZO-1 as measured by western blot analysis (**, P<0.01 vs. vehicle group; #, ##, P<0.05, 0.01 vs. H/R group).

In accordance with the *in vivo* assay, the expression level of ZO-1 was investigated. We found that ZO-1 (Figure 7) was significantly downregulated in H/R-treated cells, which was greatly reversed by 1.5 and 3 μM CEP, suggesting that the level of ZO-1 in H/R-treated bEnd.3 cells was restored by CEP.

### CEP repressed the level of VEGF-A and VEGFR2 in bEnd.3 cells

**Figure 8:**
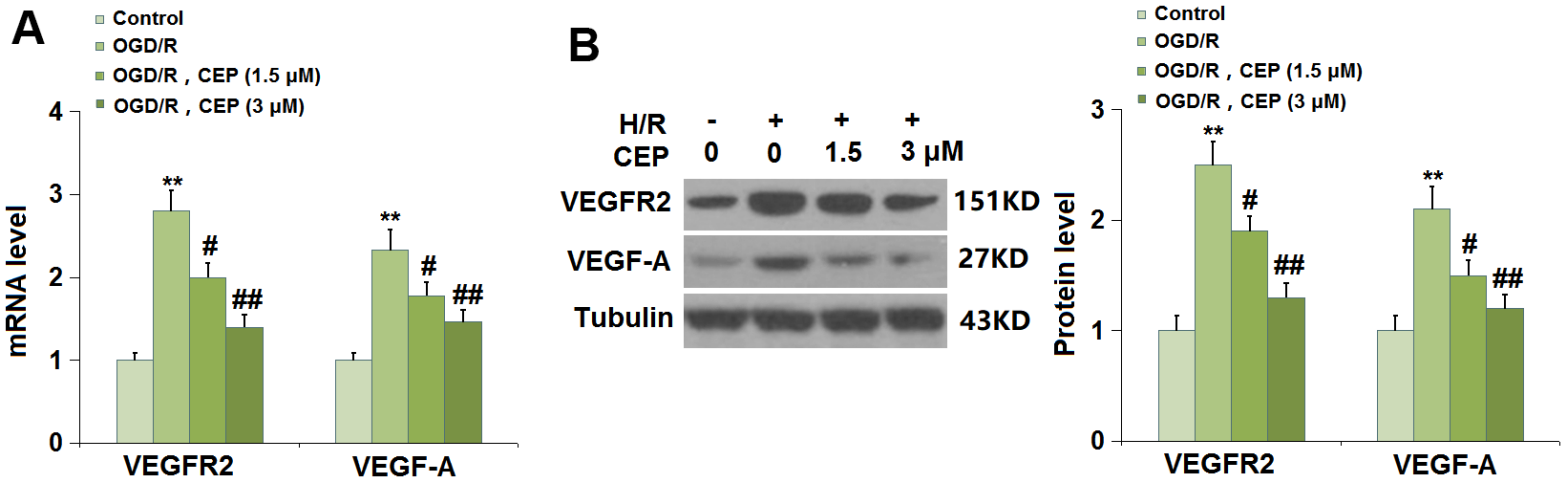
**Cepharanthine (CEP) reduced the expression of VEGF-A and VEGFR2 in brain bEND.3 endothelial cells.** (**A**) mRNA of VEGF-A and VEGFR2; (**B**) Protein of VEGF-A and VEGFR2 (**, P<0.01 vs. vehicle group; #, ##, P<0.05, 0.01 vs. H/R group).

We further evaluated the state of the VEGF-A/VEGFR2 axis in bEnd.3 cells. The expression levels of VEGF-A and VEGFR2 (Figure 8A, 8B) were found significantly elevated in H/R- challenged cells, then greatly repressed by 1.5 and 3 μM CEP, suggesting an inhibitory effect of CEP on the VEGF-A/VEGFR2 axis in H/R-challenged bEnd.3 cells.

### VEGF-A abolished the protective effects of CEP on ZO-1 level and endothelial permeability

**Figure 9:**
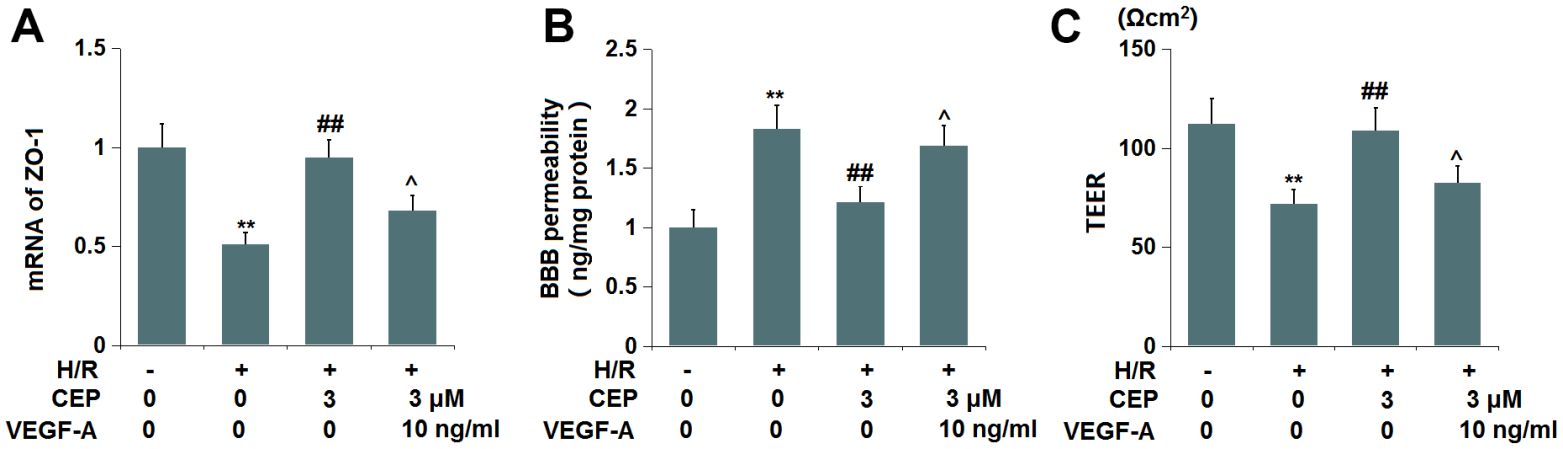
**VEGF-A abolished the protective effects of CEP on the expression of ZO-1 and endothelial permeability.** Cells were exposed to OGD/R in the presence or absence of CEP (3 μM) or VEGF-A (10 ng/ml). (**A**) mRNA of ZO-1; (**B**) Endothelial permeability was measured using FITC-dextran; (**C**) The trans-endothelial electrical resistance (TEER) (**, P<0.01 vs. vehicle group; ##, P<0.01 vs. H/R group group; ^, P<0.05 vs. H/R+CEP group).

To examine whether the function of CEP was associated with the VEGF-A/VEGFR2 axis, cells were treated with H/R, with or without CEP (3 μM) or VEGF-A (10 ng/ml). We found that the downregulation of ZO-1 in H/R-challenged cells was greatly reversed by CEP, an effect that was abolished by the co-introduction of VEGF-A (Figure 9A). Furthermore, the enhanced fluorescence intensity of FITC in H/R-treated cells was reduced by CEP, and later elevated by the co-incubation with VEGF-A (Figure 9B). Moreover, the TEER values in the control, H/R, CEP, and CEP+ VEGF-A groups were 112.6, 71.8, 108.9, and 82.5 Ωcm^2^ (Figure 9C), respectively. These results imply that the protective function of CEP on ZO-1 level and endothelial permeability was abrogated by VEGF-A.

## DISCUSSION

In the pathological process of ischemic stroke, disruption of the BBB is particularly significant. In the case of ischemic stroke complicated by diseases such as hypertension and hyperglycemia, destruction of the BBB is further aggravated after stroke due to changes in vascular anatomy and function of the central nervous system, accompanied by aggravation of nerve injury in stroke. The process of brain damage is sped up, and the risk of bleeding increased by damage to the BBB. Furthermore, cerebral edema results from BBB damage, which is the most serious disabling and fatal complication of ischemic stroke. Cerebral edema caused by ischemic stroke includes cytotoxic edema and vasogenic edema. Cerebral edema becomes progressively worse after ischemia, with cytotoxic edema occurring several minutes after an ischemic attack, and vasogenic edema occurring relatively later, which is particularly closely related to BBB damage [25, 26]. In patients with ischemic stroke, a large amount of blood-derived fluid flows into the brain parenchyma after BBB destruction, resulting in progressively increased water content in brain tissue [27]. We constructed a MCAO mice model with increased neurological scores and BBB permeability, consistent with the description by Du [28]. These pathological changes were significantly alleviated by CEP, suggesting that the neuroprotective effect of CEP on MCAO mice might be mediated by its impact on BBB permeability. Furthermore, increased BBB permeability was observed in H/R-challenged bEnd.3 cells, in accordance with the observation reported by Yang [29]. After incubation with CEP, the BBB permeability was greatly alleviated, which further evidenced the protective effect of CEP in MCAO mice.

ZO-1 is a TJ-related protein, the level change of which shows a critical impact on the structure and function of TJs. In the early stage of cerebral ischemia, the ZO-1 protein level is significantly reduced, accompanied by destroyed TJs and increased BBB permeability [30]. Changes in ZO-1 protein level are correlated with the degree of BBB damage to a certain extent, which can be used as a marker of BBB damage [31]. In the present study, ZO-1 was found downregulated in both MCAO mice and H/R- challenged bEnd.3 cells, also reported by Zhang [32]. The changes in ZO-1 protein level in both MCAO mice and H/R-challenged bEnd.3 cells were reversed by CEP, suggesting that CEP might exert its protective function by upregulating ZO-1.

VEGF is one of the main regulatory factors that increase microvascular permeability. Several studies have confirmed that the increased BBB permeability and cerebral edema can be induced by the interaction of VEGF [33, 34]. In central nervous immune diseases, hypoxia, and cerebral ischemia, the expression of VEGF in astrocytes is upregulated and induces an increase in BBB permeability [35, 36]. Furthermore, VEGF is reported to induce BBB dysfunction and increase its permeability by inducing tight junction protein degradation, occludin transport, and phosphorylation of Ser490 in occludin [37, 38]. We found that the activated VEGF-A/VEGFR2 axis in both MCAO mice and H/R-challenged bEnd.3 cells was significantly suppressed by CEP. Furthermore, the protective function of CEP on ZO-1 and endothelial permeability was extremely abrogated by VEGF-A, implying that CEP might exert its protective effect on stroke by regulating the VEGF-A/VEGFR2 axis. In future work, the mechanism will be further investigated by co-administering VEGF-A and CEP to MCAO mice.

Collectively, our data reveal that CEP maintained integrity of the BBB in stroke by mediating VEGF/VEGFR2/ZO-1 signaling.
